# Birthweight and risk markers for type 2 diabetes and cardiovascular disease in childhood: the Child Heart and Health Study in England (CHASE)

**DOI:** 10.1007/s00125-014-3474-7

**Published:** 2014-12-18

**Authors:** Claire M. Nightingale, Alicja R. Rudnicka, Christopher G. Owen, Sian L. Newton, Jennifer L. Bales, Angela S. Donin, Cathy M. McKay, Phillip J. Steer, Debbie A. Lawlor, Naveed Sattar, Derek G. Cook, Peter H. Whincup

**Affiliations:** 1 Population Health Research Institute, St George’s University of London, Cranmer Terrace, London, SW17 0RE UK; 2 Academic Department of Obstetrics and Gynaecology, Imperial College London, Chelsea and Westminster Hospital, London, UK; 3 MRC Integrative Epidemiology Unit, University of Bristol, Bristol, UK; 4 School of Social and Community Medicine, University of Bristol, Bristol, UK; 5 Institute of Cardiovascular and Medical Sciences, BHF Glasgow Cardiovascular Research Centre, University of Glasgow, Glasgow, UK

**Keywords:** Birthweight, Cardiovascular disease, Childhood, Ethnicity, Type 2 diabetes

## Abstract

**Aims/hypothesis:**

Lower birthweight (a marker of fetal undernutrition) is associated with higher risks of type 2 diabetes and cardiovascular disease (CVD) and could explain ethnic differences in these diseases. We examined associations between birthweight and risk markers for diabetes and CVD in UK-resident white European, South Asian and black African-Caribbean children.

**Methods:**

In a cross-sectional study of risk markers for diabetes and CVD in 9- to 10-year-old children of different ethnic origins, birthweight was obtained from health records and/or parental recall. Associations between birthweight and risk markers were estimated using multilevel linear regression to account for clustering in children from the same school.

**Results:**

Key data were available for 3,744 (66%) singleton study participants. In analyses adjusted for age, sex and ethnicity, birthweight was inversely associated with serum urate and positively associated with systolic BP. After additional height adjustment, lower birthweight (per 100 g) was associated with higher serum urate (0.52%; 95% CI 0.38, 0.66), fasting serum insulin (0.41%; 95% CI 0.08, 0.74), HbA_1c_ (0.04%; 95% CI 0.00, 0.08), plasma glucose (0.06%; 95% CI 0.02, 0.10) and serum triacylglycerol (0.30%; 95% CI 0.09, 0.51) but not with BP or blood cholesterol. Birthweight was lower among children of South Asian (231 g lower; 95% CI 183, 280) and black African-Caribbean origin (81 g lower; 95% CI 30, 132). However, adjustment for birthweight had no effect on ethnic differences in risk markers.

**Conclusions/interpretation:**

Birthweight was inversely associated with urate and with insulin and glycaemia after adjustment for current height. Lower birthweight does not appear to explain emerging ethnic difference in risk markers for diabetes.

**Electronic supplementary material:**

The online version of this article (doi:10.1007/s00125-014-3474-7) contains peer-reviewed but unedited supplementary material, which is available to authorised users.

## Introduction

Risks of adult chronic diseases, particularly type 2 diabetes and cardiovascular disease (CVD), appear to originate in early life, possibly before birth [1, 2]. Lower birthweight (a marker of fetal undernutrition) has been associated with increased risks of both type 2 diabetes [3] and CVD [4]. However, in many countries, ethnic minority populations have higher risks of type 2 diabetes and CVD and lower mean birthweights, raising the possibility that lower birthweight may be an important determinant of ethnic differences in risks of these diseases [5]. In the UK, South Asians have markedly higher risks of type 2 diabetes, coronary heart disease and stroke compared with white Europeans [6–8], while black African-Caribbeans have higher risks of type 2 diabetes and stroke [6–8]. These ethnic differences in type 2 diabetes and cardiovascular risks are already apparent in childhood [9, 10]. Both South Asians and black African-Caribbeans in the UK have lower mean birthweights and an increased prevalence of low birthweight compared with white Europeans [11]. However, the extent to which ethnic differences in birthweight can account for emerging ethnic differences in type 2 diabetes and CVD risks remains unknown.

We therefore examined the associations between birthweight and risk markers for type 2 diabetes and CVD at both individual and ethnic group levels in children of South Asian, black African-Caribbean and white European origin in the UK and examined the contribution of ethnic differences in birthweight to ethnic differences in these risk markers. Examining the associations between birth size and risk markers in childhood poses important analytical challenges. Childhood size is an important determinant of risk markers for type 2 diabetes and CVD and is also related to size at birth. Adjustment for current body size may affect estimates of association between birth size and disease risk markers and should attempt to estimate the longer term effect of birthweight on disease risk [12, 13]; inappropriate adjustment could be misleading [14]. Our analyses follow an earlier approach [12] in distinguishing between two components of child size, height and body fatness, which are differently associated with risk markers over the life course [15]. Because variation in birthweight or in fetal growth rate (represented by birthweight adjusted for gestational age) could be more important [3], we have analysed associations between birthweight and risk markers both with and without adjustment for gestational age.

## Methods

### Study design

The Child Heart and Health Study in England (CHASE) was a cross-sectional investigation of the cardiovascular and type 2 diabetes risk profiles of children aged 9–10 years of white European, South Asian and black African-Caribbean origin. Ethics approval was obtained from the relevant research ethics committee. Study methods have been published [9, 10, 16]. The study included 200 state primary schools in London, Birmingham and Leicester, half with a high prevalence of South Asian children (stratified by Indian, Pakistani and Bangladeshi origin) and half with a high prevalence of black African-Caribbean children (stratified by black African and black Caribbean origin). Written, informed consent was obtained for all participants and maternal permission sought to allow access to maternal health record information on the gestation and birth of the participant. Mothers were also asked to recall the hospital of birth, the birthweight of the child and whether the birth occurred on time, early or late and (if so) by how much.

### Survey measurements

Three trained research nurses measured participants between October 2004 and February 2007, each measuring approximately one-third of children in each ethnic group. Height and weight were measured and BMI calculated. Fat mass was determined from right-sided arm-to-leg bioelectrical impedance, using the Bodystat 1500 bioelectrical impedance monitor (Bodystat, Isle of Man); fat mass was derived using ethnicity- and sex-specific equations for children of this age group in the UK [17] and presented as a height-standardised fat mass index [FMI; weight (kg)/height (m)^5^], derived to be independent of height (*r* = −0.02) [17]. FMI was preferred to BMI because of its greater validity in this multiethnic population [17]. BP was measured twice in the right arm using an Omron HEM-907 (Omron Electronics, Milton Keynes, UK) [18] with an appropriate cuff size [16]. Mean systolic and diastolic BP (two readings) were adjusted for errors in BP measurement arising from use of different cuff sizes using a previously validated method [19].

A blood sample was obtained after an overnight fast. Children were asked not to eat on the morning of the examination; those who reported having eaten breakfast were excluded from analyses. Serum for an insulin assay was separated and frozen on dry ice immediately after collection. Samples were shipped to a central laboratory within 48 h. Serum insulin, plasma glucose, HbA_1c_ (measured in whole blood), serum blood lipids and serum C-reactive protein assays have previously been reported [9, 10]. Serum urate was assayed using an enzymatic method [20]. HOMA equations [21] were used to calculate insulin resistance (HOMA-IR).

### Ethnicity and socioeconomic status

Participant ethnicity was defined as previously described [10] using self-reported parental ethnicity, where available (66%), or parentally defined child ethnic origin (33%), or using information on parental and grand-parental birth place provided by the child, cross-checked with observer assessment of ethnicity (1%). Four broad ethnic categories were defined (‘white European’, ‘South Asian’, ‘black African-Caribbean’, ‘other’), with a more detailed ten-level ethnic categorisation used for adjustments (white European, Indian, Pakistani, Bangladeshi, other South Asian, other Asian, black African, black Caribbean, other black, other). Parental occupation obtained from parents or children was coded using the UK National Statistics Socioeconomic Classification (NS-SEC) for the parent with the highest classification (professional and managerial; intermediate; routine and manual; economically inactive; unclassified) [22].

### Birthweight and gestational age

Maternal medical record data on participant birthweight, gestational age and singleton/twin status were sought from several sources including: (1) the mother’s hospital records; (2) hospital birth registers; (3) the North West Thames maternity database 1988–2000 for hospital births in the former North West Thames region; (4) the Health and Social Care Information Centre for all study children born in the UK with appropriate maternal consent (provided by 92% of mothers). Data were selected preferentially from sources (1) to (4); if no information was obtained, maternal recall data were used when available.

### Statistical methods

Statistical analyses were performed using STATA/SE software (Stata/SE 12 for Windows; StataCorp LP, College Station, TX, USA). All outcome variables except systolic and diastolic BP were positively skewed and a log transformation (base *e*) was used. Birthweight was approximately normally distributed. Low birthweight was defined as a birthweight <2.5 kg [23]. Associations between birthweight and risk markers were examined, with birthweight fitted both in fifths and as a continuous variable. Departure from linearity was examined graphically (risk marker plotted against birthweight in fifths) and tested for by comparing models containing birthweight in fifths fitted either as a categorical or continuous variable. Continuous associations between birthweight and risk markers for type 2 diabetes and CVD were quantified as percentage differences (or absolute differences for BP) in each risk marker for a 100 g increase in birthweight using multilevel linear regression models adjusted for sex, age (in fourths), ethnic group (ten levels), socioeconomic position (five levels) and a random effect to allow for clustering of children within schools. The effects of additional adjustment for childhood height and FMI were examined, fitting these as continuous variables. Birthweight × height and birthweight × FMI interactions were also examined. To examine whether associations between birthweight and type 2 diabetes and CVD risk markers differed by sex or ethnic group, birthweight × sex and birthweight × ethnic group interaction terms were fitted. The effects of adjustment for gestational age and parity and of excluding mothers with gestational diabetes or parental recall of birthweight only on the associations between birthweight and risk markers were examined in sensitivity analyses.

## Results

Of 8,641 children invited to participate, 5,887 (68%) took part. Among 5,681 singleton children, 4,515 (79%) provided a fasting blood sample and had complete risk marker measurements; 3,744 (66%) also had data for birthweight, of whom 90% were born in the UK. These included 1,845 boys and 1,899 girls, 1,002 white Europeans, 1,025 South Asians, 863 black African-Caribbeans and 854 children of other ethnicity. Birthweight sources for these participants were hospital maternity records (36.1%), hospital birth register (5.6%), the North West Thames maternity database (9.1%), Health and Social Care Information Centre data (35.5%) and parental recall (13.7%).

Birthweight patterns by sex and ethnic group are shown in Table 1 and birthweight patterns by ethnic subgroup are shown in Table 1 of the electronic supplementary material (ESM). Mean birthweight was 94 g higher in boys than in girls (*p* < 0.0001), although the prevalence of low birthweight (<2.5 kg) was similar (8.0% and 8.3%, respectively). Mean birthweight was lower in all ethnic minority groups, including South Asians (particularly Indians) and black African-Caribbean groups (particularly black Caribbeans), than in white Europeans. Prevalence of low birthweight was higher among South Asians and black African-Caribbeans than among white Europeans. Birthweight was moderately correlated with current childhood height (*r* = 0.20; *p* < 0.0001) but was only weakly correlated with FMI (*r* = 0.04; *p* = 0.02).

**Table 1:** Birthweight, prevalence of low birthweight and risk makers for type 2 diabetes and CVD by sex and by ethnic group

Variable	Mean/geometric mean^a^ (95% CI) by sex		Mean/geometric mean^a^ (95% CI) by ethnic group			
	Boys (*n* = 1,845)	Girls (*n* = 1,899)	White European (*n* = 1,002)	South Asian (*n* = 1,025)	Black African-Caribbean (*n* = 863)	Other (*n* = 854)
Birthweight, g (SD)	3,289 (582)	3,195 (549)	3,345 (544)	3,114 (522)	3,264 (598)	3,250 (585)
Low birthweight (<2.5 kg), %	8.0	8.3	5.8	10.8	7.7	8.1
Age, years	9.96 (9.92, 10.00)	9.94 (9.91, 9.98)	9.96 (9.92, 10.00)	9.95 (9.91, 9.99)	9.94 (9.90, 9.98)	9.95 (9.91, 10.00)
Height, cm	140.0 (139.7, 140.3)	140.4 (140.1, 140.7)	139.2 (138.8, 139.6)	138.8 (138.3, 139.2)	143.4 (142.9, 143.8)	139.9 (139.5, 140.3)
FMI, kg/m^5a^	1.93 (1.89, 1.97)	2.18 (2.13, 2.22)	2.01 (1.96, 2.06)	2.17 (2.11, 2.23)	1.90 (1.84, 1.95)	2.13 (2.07, 2.19)
Insulin, pmol/l^a^	45.32 (43.60, 47.10)	56.57 (54.45, 58.78)	42.90 (40.94, 44.96)	56.75 (54.06, 59.57)	53.44 (50.86, 56.15)	51.13 (48.68, 53.71)
HOMA-IR^a^	0.82 (0.79, 0.86)	1.02 (0.98, 1.06)	0.78 (0.74, 0.82)	1.03 (0.98, 1.08)	0.97 (0.92, 1.02)	0.93 (0.88, 0.97)
HbA_1c_, %^a^	5.24 (5.22, 5.26)	5.24 (5.22, 5.26)	5.18 (5.15, 5.20)	5.28 (5.26, 5.31)	5.27 (5.24, 5.29)	5.22 (5.19, 5.25)
HbA_1c_, mmol/l^a^	33.50 (33.28, 33.73)	33.52 (33.30, 33.75)	32.90 (32.63, 33.17)	34.02 (33.74, 34.31)	33.82 (33.53, 34.11)	33.34 (33.05, 33.62)
Glucose, mmol/l^a^	4.55 (4.53, 4.58)	4.46 (4.44, 4.48)	4.51 (4.48, 4.53)	4.54 (4.52, 4.57)	4.47 (4.45, 4.50)	4.50 (4.47, 4.53)
Urate, mmol/l^a^	0.21 (0.21, 0.22)	0.22 (0.22, 0.23)	0.22 (0.22, 0.23)	0.22 (0.22, 0.23)	0.21 (0.20, 0.21)	0.22 (0.22, 0.23)
C-reactive protein, nmol/l^a^	4.26 (3.98, 4.55)	5.51 (5.16, 5.89)	3.93 (3.60, 4.28)	6.03 (5.51, 6.59)	4.88 (4.45, 5.36)	4.78 (4.35, 5.24)
Triacylglycerol, mmol/l^a^	0.76 (0.75, 0.78)	0.85 (0.83, 0.87)	0.80 (0.78, 0.82)	0.90 (0.88, 0.93)	0.71 (0.69, 0.73)	0.82 (0.79, 0.84)
HDL-cholesterol, mmol/l^a^	1.53 (1.52, 1.55)	1.44 (1.43, 1.46)	1.48 (1.46, 1.50)	1.45 (1.43, 1.47)	1.52 (1.50, 1.54)	1.49 (1.47, 1.51)
LDL-cholesterol, mmol/l^a^	2.62 (2.58, 2.65)	2.61 (2.58, 2.65)	2.59 (2.54, 2.63)	2.69 (2.64, 2.74)	2.55 (2.51, 2.60)	2.63 (2.58, 2.68)
Systolic BP, mmHg	105.3 (104.7, 105.9)	104.4 (103.8, 105.0)	105.0 (104.3, 105.8)	104.2 (103.4, 104.9)	104.8 (104.0, 105.5)	105.5 (104.7, 106.3)
Diastolic BP, mmHg	62.8 (62.3, 63.4)	63.1 (62.5, 63.6)	62.2 (61.5, 62.9)	63.3 (62.6, 64.0)	62.9 (62.2, 63.7)	63.4 (62.7, 64.1)

Mean and 95% CI presented for age, height and BP; birthweight levels are unadjusted; age is adjusted for sex, ethnic group and a random effect for school; all other variables are adjusted for age (in fourths), sex, ethnic group and a random effect for school

^a^Geometric mean with 95% CI presented for log-transformed variables

### Associations between birthweight and risk markers for type 2 diabetes and CVD in individuals

The continuous associations between birthweight and type 2 diabetes and CVD risk markers are shown in Table 2. Height-adjusted associations for fifths of birthweight are summarised graphically in ESM Fig. 1. The associations between birthweight and risk markers all appeared graded, with no evidence of U-shaped associations (tests for non-linearity, all *p* > 0.05). In models including adjustments for age, sex, ethnic subgroup and school, there was an inverse association between birthweight and urate and a positive association with systolic BP; there were no appreciable associations of birthweight with insulin resistance (IR), glycaemia or blood lipids (Table 2). After additional adjustment for current childhood height, birthweight had a stronger inverse association with urate and was also inversely associated with fasting insulin, HOMA-IR, HbA_1c_, fasting glucose and triacylglycerol. In continuous associations, for each 100 g lower birthweight, the largest percentage increases were observed for urate, HOMA-IR and insulin. No consistent associations were observed for C-reactive protein, LDL-cholesterol, HDL-cholesterol or BP. After separate adjustment for current childhood FMI (but without height), birthweight showed marginal inverse associations with triacylglycerol and urate but not with other risk markers. Combined adjustment for height and FMI increased the magnitude of the inverse associations observed after height adjustment between birthweight and fasting insulin, HOMA-IR, HbA_1c_, fasting glucose, urate and triacylglycerol. An inverse association between birthweight and C-reactive protein also appeared (Table 2).

**Table 2:** Associations between birthweight and risk markers for type 2 diabetes and CVD with additional adjustments for childhood height and adiposity

Blood analyte and adjustment	% Difference/difference in outcome for a 100 g increase in birthweight (95% CI)	*p* value
Insulin, pmol/l		
Standard	0.28 (−0.07, 0.65)	0.12
Standard + height	−0.41 (−0.74, −0.08)	0.02
Standard + FMI	−0.05 (−0.36, 0.27)	0.76
Standard + height + FMI	−0.72 (−1.00, −0.43)	<0.0001
HOMA-IR		
Standard	0.24 (−0.11, 0.60)	0.19
Standard + height	−0.44 (−0.76, −0.11)	0.01
Standard + FMI	−0.09 (−0.40, 0.23)	0.60
Standard + height + FMI	−0.74 (−1.02, −0.45)	<0.0001
HbA_1c_, %		
Standard	−0.03 (−0.06, 0.01)	0.12
Standard + height	−0.04 (−0.08, 0.00)	0.03
Standard + FMI	−0.04 (−0.07, 0.00)	0.05
Standard + height + FMI	−0.05 (−0.08, −0.01)	0.01
HbA_1c_, mmol/l		
Standard	−0.05 (−0.11, 0.02)	0.15
Standard + height	−0.07 (−0.13, 0.00)	0.03
Standard + FMI	−0.06 (−0.12, 0.00)	0.06
Standard + height + FMI	−0.08 (−0.14, −0.02)	0.01
Glucose, mmol/l		
Standard	−0.03 (−0.08, 0.01)	0.16
Standard + height	−0.06 (−0.10, −0.02)	0.01
Standard + FMI	−0.04 (−0.08, 0.01)	0.10
Standard + height + FMI	−0.06 (−0.11, −0.02)	0.004
Urate (mmol/l)		
Standard	−0.36 (−0.50, −0.22)	<0.0001
Standard + height	−0.52 (−0.66, −0.38)	<0.0001
Standard + FMI	−0.45 (−0.58, −0.32)	<0.0001
Standard + height + FMI	−0.61 (−0.74, −0.48)	<0.0001
C-reactive protein, nmol/l		
Standard	0.47 (−0.29, 1.29)	0.23
Standard + height	−0.23 (−0.94, 0.55)	0.56
Standard + FMI	−0.36 (−0.97, 0.29)	0.27
Standard + height + FMI	−0.99 (−1.56, −0.37)	0.002
Triacylglycerol (mmol/l)		
Standard	−0.13 (−0.34, 0.08)	0.23
Standard + height	−0.30 (−0.51, −0.09)	0.01
Standard + FMI	−0.26 (−0.46, −0.06)	0.01
Standard + height + FMI	−0.43 (−0.63, −0.23)	<0.0001
HDL-cholesterol, mmol/l		
Standard	−0.07 (−0.19, 0.04)	0.22
Standard + height	0.04 (−0.08, 0.16)	0.53
Standard + FMI	−0.01 (−0.12, 0.11)	0.89
Standard + height + FMI	0.10 (−0.01, 0.22)	0.08
LDL-cholesterol, mmol/l		
Standard	0.07 (−0.07, 0.21)	0.34
Standard + height	0.12 (−0.02, 0.27)	0.10
Standard + FMI	0.04 (−0.10, 0.18)	0.61
Standard + height + FMI	0.09 (−0.05, 0.24)	0.22
Systolic BP, mmHg^a^		
Standard	0.07 (0.01, 0.13)	0.02
Standard + height	−0.03 (−0.09, 0.03)	0.32
Standard + FMI	0.05 (−0.01, 0.11)	0.12
Standard + height + FMI	−0.05 (−0.11, 0.00)	0.07
Diastolic BP, mmHg^a^		
Standard	0.02 (−0.03, 0.08)	0.43
Standard + height	−0.02 (−0.07, 0.04)	0.55
Standard + FMI	0.00 (−0.05, 0.05)	0.96
Standard + height + FMI	−0.04 (−0.09, 0.02)	0.18

Percentage differences in outcome are presented for log-transformed variables (all except BP); standard adjustment is for sex, age (in fourths), NS-SEC group, ethnic subgroup and a random effect for school

^a^Absolute differences in BP are presented

The associations between birthweight and risk markers in models adjusted for height are shown separately by sex and by ethnic group with tests for interaction (ESM Table 2). Associations between birthweight and IR makers were stronger in girls compared with boys and in white Europeans compared with other ethnic groups. None of the birthweight–risk marker associations showed evidence of statistical interactions between birthweight and height or between birthweight and FMI (all *p* > 0.05; ESM Table 2). Associations between birthweight in fifths and risk markers are shown in ESM Table 3 to allow for the possibility of non-linearity in these associations. This showed similar patterns to the continuous associations presented in Table 2.

Associations between birthweight and risk markers with cumulative adjustments for gestational age and parity are shown in ESM Table 4. Associations between birthweight and fasting insulin, HOMA-IR and urate were little affected by gestational age adjustment; the association with HbA_1c_ was strengthened and those with fasting glucose and triacylglycerol were weakened by approximately one-third. Additional adjustment for parity did not appreciably affect associations between birthweight and risk markers. Exclusion of 94 participants born to mothers with gestational diabetes or 512 participants for whom birthweight data were based on parental recall (rather than medical records) had no material effect on these associations (ESM Table 5).

### Ethnic differences in risk markers for type 2 diabetes and CVD: the contribution of birthweight

Ethnic differences in risk markers for type 2 diabetes and CVD in this study population have previously been reported [9, 10]. Ethnic patterns in risk markers in the 3,744 singleton children with birthweight data are shown in Table 1 and ethnic differences in risk markers for CVD are shown in Table 3 (South Asians–white Europeans) and Table 4 (black African-Caribbeans–white Europeans). Compared with white Europeans, South Asians had similar mean age and height. Their mean FMI, fasting insulin, HOMA-IR, HbA_1c_, fasting glucose, C-reactive protein, triacylglycerol, LDL-cholesterol and diastolic BP were higher; mean HDL-cholesterol was lower. Compared with white Europeans, black African-Caribbeans were taller and had a lower mean FMI. They had higher mean levels of fasting insulin, HOMA-IR, HbA_1c_, HDL-cholesterol and diastolic BP, and lower mean levels of fasting glucose, urate, triacylglycerol and systolic BP.

**Table 3:** Ethnic differences between South Asians and white Europeans in risk markers for type 2 diabetes and CVD adjusted for height: effect of adjustment for birthweight

Blood analyte	Adjustment for birthweight	South Asian − white European			South Asian subgroup								
					Indian − white European			Pakistani − white European			Bangladeshi − white European		
		% Difference/difference	95% CI	*p* value	% Difference/difference	95% CI	*p* value	% Difference/difference	95% CI	*p* value	% Difference/difference	95% CI	*p* value
Insulin, pmol/l	No	33.37	26.36, 40.77	<0.0001	33.17	23.34, 43.79	<0.0001	23.74	14.90, 33.27	<0.0001	53.63	40.80, 67.63	<0.0001
	Yes	32.19	25.18, 39.58	<0.0001	31.80	22.02, 42.36	<0.0001	22.69	13.89, 32.17	<0.0001	52.61	39.85, 66.54	<0.0001
HOMA-IR	No	32.89	25.97, 40.20	<0.0001	32.10	22.44, 42.53	<0.0001	23.92	15.14, 33.37	<0.0001	53.19	40.51, 67.01	<0.0001
	Yes	31.63	24.72, 38.93	<0.0001	30.64	21.03, 41.01	<0.0001	22.79	14.06, 32.19	<0.0001	52.09	39.49, 65.83	<0.0001
HbA_1c_, %	No	2.08	1.50, 2.66	<0.0001	2.71	1.88, 3.54	<0.0001	2.63	1.83, 3.44	<0.0001	0.71	−0.21, 1.64	0.13
	Yes	1.99	1.41, 2.58	<0.0001	2.60	1.77, 3.44	<0.0001	2.55	1.75, 3.36	<0.0001	0.64	−0.28, 1.57	0.17
HbA_1c_, mmol/l	No	3.49	2.47, 4.52	<0.0001	4.57	3.10, 6.05	<0.0001	4.48	3.06, 5.92	<0.0001	1.06	−0.55, 2.69	0.20
	Yes	3.34	2.31, 4.38	<0.0001	4.39	2.92, 5.88	<0.0001	4.34	2.92, 5.78	<0.0001	0.95	−0.66, 2.58	0.25
Glucose, mmol/l	No	0.82	0.11, 1.53	0.02	1.11	0.10, 2.13	0.03	1.01	0.03, 1.99	0.04	1.26	0.12, 2.42	0.03
	Yes	0.69	−0.02, 1.41	0.06	0.96	−0.05, 1.98	0.06	0.89	−0.09, 1.87	0.08	1.17	0.02, 2.33	0.05
Urate, mmol/l	No	1.08	−1.12, 3.34	0.34	−2.17	−5.19, 0.94	0.17	−1.47	−4.40, 1.55	0.34	8.84	5.09, 12.74	<0.0001
	Yes	−0.07	−2.26, 2.16	0.95	−3.47	−6.45, −0.40	0.03	−2.57	−5.47, 0.41	0.09	7.80	4.09, 11.65	<0.0001
C-reactive protein, nmol/l	No	55.06	37.98, 74.25	<0.0001	43.79	21.74, 69.84	<0.0001	67.76	42.87, 96.98	<0.0001	56.57	29.81, 88.83	<0.0001
	Yes	54.21	37.07, 73.50	<0.0001	42.97	20.91, 69.06	<0.0001	66.95	42.06, 96.20	<0.0001	55.94	29.22, 88.17	<0.0001
Triacylglycerol, mmol/l	No	13.50	9.68, 17.45	<0.0001	11.16	5.88, 16.70	<0.0001	13.54	8.32, 19.00	<0.0001	19.37	12.98, 26.13	<0.0001
	Yes	12.73	8.91, 16.69	<0.0001	10.31	5.04, 15.84	<0.0001	12.82	7.62, 18.27	<0.0001	18.76	12.38, 25.49	<0.0001
HDL-cholesterol, mmol/l	No	−2.14	−3.90, −0.34	0.02	−0.13	−2.69, 2.49	0.92	−1.08	−3.53, 1.42	0.39	−6.26	−8.96, −3.49	<0.0001
	Yes	−2.06	−3.84, −0.25	0.03	−0.04	−2.61, 2.60	0.98	−1.00	−3.46, 1.52	0.43	−6.20	−8.91, −3.42	<0.0001
LDL-cholesterol, mmol/l	No	3.83	1.52, 6.20	0.001	3.87	0.60, 7.26	0.02	4.70	1.50, 7.99	0.004	1.67	−1.95, 5.42	0.37
	Yes	4.08	1.73, 6.47	<0.001	4.19	0.88, 7.60	0.01	4.96	1.74, 8.28	0.002	1.88	−1.76, 5.65	0.32
Systolic BP, mmHg^a^	No	−0.67	−1.59, 0.25	0.15	−0.47	−1.78, 0.84	0.48	−1.02	−2.28, 0.25	0.12	−0.35	−1.84, 1.13	0.64
	Yes	−0.74	−1.67, 0.18	0.12	−0.55	−1.87, 0.77	0.41	−1.08	−2.35, 0.19	0.10	−0.40	−1.89, 1.08	0.59
Diastolic BP, mmHg^a^	No	1.19	0.34, 2.04	0.01	1.76	0.54, 2.97	0.005	0.45	−0.72, 1.62	0.45	1.48	0.10, 2.85	0.04
	Yes	1.15	0.29, 2.01	0.01	1.72	0.50, 2.94	0.01	0.41	−0.76, 1.59	0.49	1.45	0.07, 2.83	0.04

All differences are adjusted for sex, age (in fourths), NS-SEC group, height and a random effect for school

^a^Absolute differences in BP are presented; percentage differences in outcome are presented for log-transformed variables (all except BP)

**Table 4:** Ethnic differences between black African-Caribbeans and white Europeans in risk markers for type 2 diabetes and CVD adjusted for height: effect of adjustment for birthweight

Blood analyte	Adjustment for birthweight	Black African-Caribbean − white European			Black African-Caribbean subgroups					
					Black Caribbean − white European			Black African − white European		
		% Difference/difference	95% CI	*p* value	% Difference/difference	95% CI	*p* value	% Difference/difference	95% CI	*p* value
Insulin, pmol/l	No	10.55	4.52, 16.93	<0.001	16.13	8.01, 24.87	<0.0001	7.45	0.37, 15.02	0.04
	Yes	9.91	3.90, 16.27	<0.001	15.20	7.12, 23.89	<0.001	7.05	0.00, 14.60	0.05
HOMA-IR	No	10.36	4.39, 16.67	<0.001	15.88	7.84, 24.51	<0.0001	7.51	0.50, 15.02	0.04
	Yes	9.68	3.74, 15.96	0.001	14.88	6.89, 23.47	<0.001	7.09	0.10, 14.57	0.05
HbA_1c_, %	No	1.53	0.93, 2.13	<0.0001	1.39	0.62, 2.17	<0.001	1.75	1.02, 2.49	<0.0001
	Yes	1.47	0.87, 2.07	<0.0001	1.31	0.54, 2.10	<0.001	1.72	0.99, 2.45	<0.0001
HbA_1c_, mmol/l	No	2.45	1.40, 3.51	<0.0001	2.20	0.84, 3.58	0.001	2.84	1.56, 4.14	<0.0001
	Yes	2.35	1.30, 3.42	<0.0001	2.07	0.71, 3.45	0.003	2.78	1.50, 4.08	<0.0001
Glucose, mmol/l	No	−1.16	−1.89, −0.44	0.002	−0.96	−1.89, −0.01	0.05	−0.88	−1.76, 0.01	0.05
	Yes	−1.24	−1.97, −0.51	<0.001	−1.07	−2.01, −0.12	0.03	−0.93	−1.81, −0.05	0.04
Urate, mmol/l	No	−8.84	−10.94, −6.69	<0.0001	−6.31	−9.11, −3.43	<0.0001	−10.68	−13.17, −8.12	<0.0001
	Yes	−9.52	−11.61, −7.39	<0.0001	−7.30	−10.06, −4.46	<0.0001	−11.14	−13.61, −8.60	<0.0001
C-reactive protein, nmol/l	No	10.50	−2.30, 24.97	0.11	21.16	3.22, 42.23	0.02	9.05	−6.13, 26.69	0.26
	Yes	10.11	−2.69, 24.59	0.13	20.62	2.68, 41.70	0.02	8.81	−6.35, 26.44	0.27
Triacylglycerol, mmol/l	No	−13.02	−16.07, −9.85	<0.0001	−9.94	−14.02, −5.67	<0.0001	−15.27	−18.87, −11.50	<0.0001
	Yes	−13.39	−16.44, −10.23	<0.0001	−10.48	−14.54, −6.21	<0.0001	−15.50	−19.09, −11.75	<0.0001
HDL-cholesterol, mmol/l	No	4.32	2.34, 6.34	<0.0001	3.33	0.79, 5.93	0.01	4.62	2.21, 7.09	<0.001
	Yes	4.38	2.39, 6.40	<0.0001	3.40	0.85, 6.02	0.01	4.66	2.25, 7.13	<0.001
LDL-cholesterol, mmol/l	No	−0.44	−2.76, 1.94	0.71	3.58	0.46, 6.80	0.02	−3.77	−6.49, −0.97	0.01
	Yes	−0.29	−2.62, 2.10	0.81	3.83	0.68, 7.07	0.02	−3.66	−6.39, −0.86	0.01
Systolic BP, mmHg^a^	No	−2.04	−3.00, −1.08	<0.0001	−1.39	−2.64, −0.14	0.03	−2.68	−3.85, −1.51	<0.0001
	Yes	−2.08	−3.05, −1.12	<0.0001	−1.45	−2.70, −0.19	0.02	−2.70	−3.88, −1.53	<0.0001
Diastolic BP, mmHg^a^	No	0.07	−0.81, 0.96	0.87	−0.07	−1.22, 1.08	0.91	0.13	−0.95, 1.21	0.82
	Yes	0.05	−0.84, 0.94	0.91	−0.10	−1.26, 1.06	0.86	0.11	−0.97, 1.20	0.84

All differences are adjusted for sex, age (in fourths), NS-SEC group, height and a random effect for school

^a^Absolute differences in BP are presented; percentage differences in outcome are presented for log-transformed variables (all except BP)

The effects of birthweight adjustment on ethnic differences in risk markers between South Asians, South Asian subgroups (Indian, Pakistani, Bangladeshi) and white Europeans in height-standardised models are shown in Table 3. In comparisons with white Europeans, the markedly higher mean levels of fasting insulin, HOMA-IR, HbA_1c_, glucose, C-reactive protein, triacylglycerol and diastolic BP among South Asians were minimally affected by birthweight adjustment (being reduced by between 14% for fasting glucose and 2% for C-reactive protein); the lower HDL-cholesterol levels in South Asians were little affected. Birthweight adjustment had similarly little impact on the magnitude of ethnic differences in the South Asian subgroups, including the larger differences in fasting insulin, urate, triacylglycerol and HDL-cholesterol for Bangladeshis.

The effects of birthweight adjustment on ethnic differences in risk markers between black African-Caribbeans (and black Africans and black Caribbeans separately) and white Europeans in height–standardised models are shown in Table 4. In comparison with white Europeans, the markedly higher mean levels of fasting insulin, HOMA-IR, HbA_1c_ and HDL-cholesterol in black African-Caribbeans were largely unaffected by birthweight adjustment; the lower mean levels of urate, triacylglycerol and systolic BP were similarly little affected by birthweight adjustment. Adjustment for birthweight similarly had little impact on the magnitude of ethnic differences in the separate black African-Caribbean subgroups, including the larger differences in fasting insulin in black Caribbeans and the lower urate, triacylglycerol and systolic BP levels in black Africans. In parallel analyses without height adjustment (ESM Tables 6 and 7 for South Asians and black Africans, respectively), similar results were observed; birthweight adjustment had very little effect on ethnic differences in risk markers. Repeating these analyses fitting birthweight as a dichotomous variable (<2.5 kg, ≥2.5 kg) did not materially affect the results (data available from authors).

## Discussion

In this multiethnic study of prepubertal children, birthweight was associated with some risk markers for type 2 diabetes and CVD, although these were in most cases dependent on adjustment for childhood size (particularly height). In analyses unadjusted for childhood size, birthweight was inversely associated with serum urate and positively associated with systolic BP but not notably related to other risk markers. After adjustment for childhood height, birthweight was inversely associated not only with urate but also with fasting insulin, HOMA-IR, HbA_1c_, fasting glucose and triacylglycerol. Further adjustment for FMI strengthened many of these height-adjusted associations. The associations between birthweight, fasting insulin and HOMA-IR tended to be stronger in girls and in white Europeans; associations with other risk markers were mostly consistent across sexes and ethnic groups. The associations were little affected by gestational age, parity adjustment or exclusion of mothers with gestational diabetes. There were marked ethnic differences in type 2 diabetes risk markers, with IR being higher among South Asians and to a lesser extent black African-Caribbeans compared with white Europeans, while mean birthweight was lower among South Asians and to a lesser extent black African-Caribbeans. However, adjustment for differences in birthweight did not account for ethnic differences in risk markers.

### Relation to previous studies

In the present study, birthweight was inversely associated with IR and its correlates (including triacylglycerol and urate). This is consistent with the results of most population-based studies including 250+ prepubertal children [6, 12, 13, 24–26], though not all [27, 28]. As in most studies reporting an inverse association between birthweight and IR, this was only apparent after adjustment for current size [13, 24]. In the present study, as in an earlier report [12], adjustment for height alone produced the inverse birthweight–IR association, although adjustment for body fatness alone did not reveal the association. An inverse birthweight–IR association has previously been reported in white Europeans [12, 13], in Indians [24] and in African-origin populations [26]. No previous studies have compared the strength of birthweight–IR associations among South Asians and white Europeans, although in the Bogalusa Heart Study this association was slightly stronger among African-Americans than among whites [26]. The inverse association, after adjustment for current size, between birthweight and circulating glucose levels (both fasting glucose and HbA_1c_) observed in the present study has been widely reported in adults [3] but has been reported in only one previous study in children [25], with a further study reporting an inverse association for ponderal index at birth [28]. Several other relevant studies reported null associations [13, 24, 27]. However, positive birthweight–glucose associations have been reported in Aboriginal child populations [13, 29] as in adults [30]. The inverse association detected in the present study may reflect the greater size and statistical power of the present study compared with previous investigations. The inverse association observed between birthweight and circulating urate (apparent without adjustment for height in the present study) is consistent with several earlier reports [27, 31, 32]. Although those reports tended to emphasise the role of urate in developing hypertension [31, 32], in the present study urate was associated with fasting insulin (*r* = 0.21) and only weakly with systolic or diastolic BP (*r* = 0.11 and 0.08, respectively), although a large Mendelian randomisation study suggested that urate had no causal role in the development of type 2 diabetes [33]. Despite several previous reports suggesting that birthweight is inversely associated with childhood BP after adjustment for current size [34], we observed no inverse associations between birthweight and BP in the present study, even after adjustment for current size. The absence of any appreciable association between birthweight and LDL-cholesterol is consistent with previous systematic review evidence based on studies both in children and adults [35].

Although fetal undernutrition could contribute to ethnic differences in risks of type 2 diabetes and CVD (especially the high risks in South Asians) [5], this is to our knowledge the first study to examine the contribution of birthweight to ethnic differences in relevant risk markers. The absence of an appreciable contribution of birthweight (or the presence of low birthweight, <2.5 kg) to South Asian/white European differences in markers of IR (despite a mean difference of ~250 g in birthweight between these groups) is consistent with earlier reports in adults suggesting that a 250 g difference in birthweight would only explain a 7–10% difference in type 2 diabetes risk [3], rather than the three- to fourfold difference actually observed [6].

### Strengths and limitations

Strengths of this study include its large sample size and balanced representation of children of South Asian (including Indian, Pakistani, Bangladeshi), black African-Caribbean (including black African, black Caribbean) and white European origin from three major UK cities; the measurement of important early risk markers for type 2 diabetes (particularly fasting glucose, HbA_1c_ and markers of IR including fasting insulin, HOMA-IR and urate [36]; and the measurement of important early risk markers for CVD (particularly LDL-cholesterol and BP). Although the study response rate was only moderate, mean birthweight levels and low birthweight prevalences among the different ethnic groups in the present study were similar to those in national data [11], suggesting that the study population was substantially representative for the key exposure studied. FMI derived from bioelectrical impedance and used with validated ethnicity- and sex-specific equations [17] provided a robust marker of body fatness in this multiethnic population [17]. Although the device used to measure BP (Omron HEM-907) has not been validated in children, validation studies in adults showed that the performance of the instrument was similar at different BP levels [18]. Several data sources were used to obtain contemporaneously recorded birthweight data from health records. Parental recall data (which showed very close agreement with health record data in 1,779 participants with birthweight data from both sources) were available for a further 14% of participants. Study participants with birthweight data (83%) had similar risk marker profiles to those of participants without birthweight data. Birthweight patterns observed among study participants (including both sex and ethnic differences) corresponded closely with those for the UK overall [11]. In analyses of associations between birthweight, diabetes and cardiovascular risk, we explored the impact of specific components of current body size and were able to make adjustments for potential confounders including gestational age, socioeconomic status, parity and gestational diabetes. However, this study was not able to capture ethnic differences in body composition at birth other than those in birthweight; recent reports have suggested that South Asian infants in the UK have higher fat mass and differences are not well captured by birthweight [37].

### Implications

Our results suggest that lower birthweight is associated with higher levels of IR and circulating glucose concentrations in this multiethnic childhood population, though not with cardiovascular risk markers. With the exception of urate, these associations appear to be dependent on adjustment for current height (though not on current body fatness, a measure which is completely independent of height). Such adjusted models effectively attempt to estimate the longer term effect of lower birthweight on disease risk while standardising for current height [12], and may reflect the growth trajectories taken to reach current body size [38]. Such standardisation for childhood height appears reasonable: height is strongly positively associated with IR and its correlates during childhood, but the association is attenuated, or reversed, by early adult life [12, 13]. Conversely, body fatness is positively associated with IR and its correlates in adulthood as well as in childhood [39], and in the present study it did not appear to be a confounder in models unadjusted for height. Thus, standardisation of birthweight–risk marker associations for current body fatness may be inappropriate and represent overadjustment [15]. The associations between birthweight, IR and glycaemia, though varying by sex and ethnic group, are graded, not appreciably altered by adjustment for observed confounders and consistent with the findings of other studies, suggesting that there may be an important underlying causal association with potential implications for type 2 diabetes prevention. Defining the strength of the underlying causal associations is important for assessing both causality and the preventive potential of birthweight modification. Based on the observed associations, a potentially feasible increase in birthweight (~100 g) could reduce IR by ~0.4% and glucose concentrations by 0.04–0.06%. These associations are small; substantially greater reductions could potentially be obtained by moderate reductions in childhood body fatness [39] or energy intake [40]. However, such interpretation depends on the underlying causal association, as low birthweight could denote a considerably stronger and potentially important association between early nutrition and subsequent disease risk [5, 12]. It is also possible that strategies to improve fetal nutrition, for example through maternal nutritional supplementation, could benefit offspring health independently of birthweight [41].

Previous reports have raised the possibility that low birthweight, as a marker of fetal undernutrition, could help to explain ethnic differences in type 2 diabetes and cardiovascular risk [5, 10]. However, the results of the present study suggest that ethnic differences in birthweight do not make an important contribution to explaining ethnic differences in IR and glycaemia between South Asians, black African-Caribbeans and white Europeans. This particularly reflects the small sizes of the associations between birthweight, insulin and glycaemia in individual participants, so that adjustment for the observed ethnic differences in mean birthweight (more than 200 g between South Asians and white Europeans) had little effect on ethnic differences in IR and glycaemia. However, this does not exclude the possibility that other factors operating in utero or at birth, including maternal nutrition and maternal glucose control [42] and body composition at birth (e.g. body fatness [37]), could be important determinants of ethnic differences in diabetes or cardiovascular risk. However, in seeking the causes of high emerging type 2 diabetes risks among South Asians and black African-Caribbeans in the UK, it will also be important to consider the later influences of childhood overweight, nutrition (including energy intake), physical activity and fitness [39, 40, 43].

## Electronic supplementary material

Below is the link to the electronic supplementary material.

ESM Table 1 (PDF 38 kb)

ESM Table 2 (PDF 43 kb)

ESM Table 3 (PDF 52 kb)

ESM Table 4 (PDF 42 kb)

ESM Table 5 (PDF 42 kb)

ESM Table 6 (PDF 45 kb)

ESM Table 7 (PDF 44 kb)

ESM Fig. 1 (PDF 47 kb)

ESM Acknowledgments (PDF 63 kb)

## Abbreviations

CHASE: Child Heart and Health Study in England
CVD: Cardiovascular disease
FMI: Fat mass index
IR: Insulin resistance
NS-SEC: National Statistics Socioeconomic Classification
