# Supplementary material for: Birthweight and risk markers for type 2 diabetes and cardiovascular disease in childhood: the Child Heart and Health Study in England (CHASE)
Source: Diabetologia. 2014 Dec 18;58(3):474–84. doi: 10.1007/s00125-014-3474-7 (PMC4320299; doi:10.1007/s00125-014-3474-7)
Supplement: Supplementary file 1 — (PDF 38 kb) [file 125_2014_3474_MOESM1_ESM.pdf]

ESM Table 1: Birth weight by ethnic sub-categories

	Birth weight (g)			
Ethnic sub-group	N	Mean	SD	% low BW (<2.5 kg)
White European	1002	3345	544	5.8
Indian	324	3091	521	11.1
Pakistani	363	3127	537	11.6
Bangladeshi	252	3118	480	8.3
South Asian other	86	3134	585	14.0
Asian other	198	3165	598	11.1
Black African	446	3308	586	7.4
Black Caribbean	355	3206	620	8.5
Black other	62	3288	534	4.8
Other	656	3275	579	7.2
All	3744	3242	567	8.1

Abbreviations: BW, birth weight.
