# Supplementary material for: Birthweight and risk markers for type 2 diabetes and cardiovascular disease in childhood: the Child Heart and Health Study in England (CHASE)
Source: Diabetologia. 2014 Dec 18;58(3):474–84. doi: 10.1007/s00125-014-3474-7 (PMC4320299; doi:10.1007/s00125-014-3474-7)
Supplement: Supplementary file 2 — (PDF 43 kb) [file 125_2014_3474_MOESM2_ESM.pdf]

ESM Table 2: Associations between risk markers for type 2 diabetes and cardiovascular disease and birth weight adjusted for childhood height: by sex and by ethnic group

Outcome	% Difference/difference for 100g increase in birth weight (95% CI)								
	Boys (n = 1845)	Girls (n = 1899)	p (Int. BW & sex)	white European (n = 1002)	South Asian (n = 1025)	Black African-Caribbean (n = 863)	p (Int. BW & EG)	p (Int. BW & Height)	p (Int. BW & FMI)
Insulin (pmol/l)	-0.11 (-0.56, 0.37)	-0.76 (-1.19, -0.30)	0.04	-0.79 (-1.39, -0.15)	-0.58 (-1.22, 0.09)	0.43 (-0.24, 1.14)	0.02	0.81	0.06
HOMA-IR	-0.15 (-0.60, 0.32)	-0.77 (-1.20, -0.31)	0.05	-0.83 (-1.42, -0.20)	-0.63 (-1.25, 0.04)	0.42 (-0.23, 1.13)	0.01	0.77	0.09
HbA1c (%)	-0.08 (-0.13, -0.03)	0.00 (-0.05, 0.05)	0.02	-0.08 (-0.15, -0.01)	0.00 (-0.07, 0.07)	-0.01 (-0.08, 0.05)	0.25	0.68	0.38
HbA1c (mmol/l)	-0.14 (-0.22, -0.05)	0.01 (-0.08, 0.10)	0.02	-0.14 (-0.26, -0.02)	0.00 (-0.13, 0.13)	-0.02 (-0.14, 0.10)	0.22	0.63	0.36
Glucose (mmol/l)	-0.06 (-0.12, 0.00)	-0.06 (-0.12, 0.00)	0.96	-0.04 (-0.13, 0.05)	-0.11 (-0.20, -0.02)	-0.03 (-0.12, 0.05)	0.46	0.75	0.09
Urate (mmol/l)	-0.46 (-0.64, -0.27)	-0.60 (-0.78, -0.40)	0.31	-0.69 (-0.95, -0.42)	-0.44 (-0.71, -0.15)	-0.55 (-0.81, -0.29)	0.43	0.09	0.15
C-reactive protein (nmol/l)	-0.66 (-1.58, 0.36)	0.20 (-0.84, 1.37)	0.24	-0.83 (-2.12, 0.66)	1.15 (-0.46, 3.02)	-1.01 (-2.24, 0.41)	0.09	0.13	0.54
Triacylglycerol (mmol/l)	-0.32 (-0.61, -0.03)	-0.31 (-0.61, -0.01)	0.95	-0.51 (-0.92, -0.10)	-0.54 (-0.95, -0.11)	0.10 (-0.32, 0.53)	0.06	0.88	0.43
HDL-cholesterol (mmol/l)	0.07 (-0.09, 0.23)	0.00 (-0.16, 0.17)	0.55	0.37 (0.14, 0.62)	-0.13 (-0.36, 0.11)	-0.17 (-0.39, 0.05)	0.001	0.02	0.82
LDL-cholesterol (mmol/l)	0.08 (-0.11, 0.28)	0.15 (-0.06, 0.36)	0.66	0.13 (-0.15, 0.43)	-0.04 (-0.33, 0.26)	0.04 (-0.23, 0.33)	0.70	0.18	0.07
Systolic BP (mmHg)[a]	-0.05 (-0.13, 0.03)	-0.02 (-0.10, 0.06)	0.63	-0.02 (-0.13, 0.10)	0.03 (-0.09, 0.15)	-0.08 (-0.19, 0.03)	0.43	0.43	0.77
Diastolic BP (mmHg)[a]	-0.05 (-0.12, 0.03)	0.02 (-0.06, 0.09)	0.21	0.03 (-0.08, 0.14)	0.02 (-0.09, 0.13)	0.00 (-0.11, 0.10)	0.91	0.41	0.17

[a] Absolute differences in blood pressure are presented in mmHg.
Percentage differences in outcome are presented for log transformed variables (all except blood pressure).
All models adjusted for age (in fourths), sex, ethnic group, NS-SEC group, height, an interaction between birth weight and sex for estimates by sex or an interaction between birth weight and ethnic group for estimates by ethnic group and a random effect for school. Birth weight, height and fat mass index were all fitted as continuous variables.
Abbreviations: BP, blood pressure; CI, confidence interval; Int., Interaction.
