# Supplementary material for: Birthweight and risk markers for type 2 diabetes and cardiovascular disease in childhood: the Child Heart and Health Study in England (CHASE)
Source: Diabetologia. 2014 Dec 18;58(3):474–84. doi: 10.1007/s00125-014-3474-7 (PMC4320299; doi:10.1007/s00125-014-3474-7)
Supplement: Supplementary file 3 — (PDF 52 kb) [file 125_2014_3474_MOESM3_ESM.pdf]

ESM Table 3: Risk markers for type 2 diabetes and cardiovascular disease by fifths of birth weight with additional adjustments for childhood height and adiposity

	BW group (fifths)	Geometric mean/mean (95% confidence interval), p-value for trend							
		Basic adjustments		Basic + Height		Basic + FMI		Basic + Height + FMI	
Insulin (pmol/l)	1 Highest BW	50.28 (47.76, 52.93)		47.39 (45.09, 49.81)		49.73 (47.43, 52.16)		46.90 (44.80, 49.10)	
	2	52.41 (49.73, 55.23)		50.82 (48.32, 53.46)		51.42 (48.99, 53.98)		49.89 (47.62, 52.26)	
	3	51.95 (49.40, 54.64)		51.80 (49.35, 54.38)		52.03 (49.66, 54.51)		51.88 (49.61, 54.25)	
	4	49.39 (46.93, 51.99)		51.03 (48.57, 53.61)		49.47 (47.19, 51.87)		51.10 (48.84, 53.48)	
	5 Lowest BW	49.64 (47.15, 52.26)	0.25	52.70 (50.14, 55.39)	0.002	51.05 (48.68, 53.54)	0.83	54.18 (51.75, 56.72)	<0.0001
HOMA-IR	1 Highest BW	0.91 (0.86, 0.95)		0.86 (0.82, 0.90)		0.90 (0.86, 0.94)		0.85 (0.81, 0.89)	
	2	0.95 (0.90, 1.00)		0.92 (0.88, 0.97)		0.93 (0.89, 0.98)		0.90 (0.86, 0.95)	
	3	0.94 (0.90, 0.99)		0.94 (0.90, 0.99)		0.95 (0.90, 0.99)		0.94 (0.90, 0.99)	
	4	0.90 (0.85, 0.94)		0.92 (0.88, 0.97)		0.90 (0.86, 0.94)		0.93 (0.89, 0.97)	
	5 Lowest BW	0.90 (0.86, 0.95)	0.35	0.96 (0.91, 1.01)	0.001	0.93 (0.89, 0.97)	0.66	0.98 (0.94, 1.03)	<0.0001
HbA1c (%)	1 Highest BW	5.21 (5.19, 5.24)		5.21 (5.18, 5.23)		5.21 (5.18, 5.24)		5.20 (5.18, 5.23)	
	2	5.25 (5.22, 5.28)		5.25 (5.22, 5.28)		5.25 (5.22, 5.28)		5.25 (5.22, 5.27)	
	3	5.23 (5.20, 5.25)		5.23 (5.20, 5.25)		5.23 (5.20, 5.25)		5.23 (5.20, 5.25)	
	4	5.26 (5.23, 5.28)		5.26 (5.23, 5.29)		5.26 (5.23, 5.28)		5.26 (5.23, 5.29)	
	5 Lowest BW	5.24 (5.21, 5.26)	0.13	5.24 (5.22, 5.27)	0.03	5.24 (5.21, 5.27)	0.07	5.25 (5.22, 5.27)	0.01
HbA1c (mmol/l)	1 Highest BW	33.25 (32.95, 33.55)		33.19 (32.89, 33.49)		33.23 (32.94, 33.53)		33.17 (32.87, 33.47)	
	2	33.68 (33.37, 33.99)		33.64 (33.34, 33.95)		33.65 (33.34, 33.96)		33.62 (33.31, 33.93)	
	3	33.43 (33.14, 33.73)		33.43 (33.14, 33.72)		33.43 (33.14, 33.73)		33.43 (33.14, 33.72)	
	4	33.73 (33.43, 34.03)		33.76 (33.47, 34.07)		33.73 (33.43, 34.03)		33.76 (33.46, 34.07)	
	5 Lowest BW	33.51 (33.21, 33.81)	0.16	33.58 (33.28, 33.88)	0.04	33.55 (33.25, 33.85)	0.09	33.61 (33.31, 33.91)	0.02
Glucose (mmol/l)	1 Highest BW	4.49 (4.46, 4.52)		4.48 (4.45, 4.51)		4.49 (4.46, 4.52)		4.48 (4.45, 4.51)	
	2	4.52 (4.49, 4.55)		4.52 (4.49, 4.55)		4.52 (4.49, 4.55)		4.52 (4.49, 4.55)	
	3	4.50 (4.47, 4.53)		4.50 (4.47, 4.53)		4.50 (4.47, 4.53)		4.50 (4.47, 4.53)	
	4	4.50 (4.47, 4.53)		4.51 (4.48, 4.53)		4.50 (4.47, 4.53)		4.51 (4.48, 4.53)	
	5 Lowest BW	4.53 (4.50, 4.56)	0.15	4.54 (4.51, 4.57)	0.006	4.53 (4.50, 4.56)	0.11	4.54 (4.51, 4.57)	0.004
Urate (mmol/l)	1 Highest BW	0.21 (0.21, 0.22)		0.21 (0.21, 0.21)		0.21 (0.21, 0.22)		0.21 (0.21, 0.21)	
	2	0.22 (0.21, 0.22)		0.21 (0.21, 0.22)		0.21 (0.21, 0.22)		0.21 (0.21, 0.22)	
	3	0.22 (0.22, 0.22)		0.22 (0.22, 0.22)		0.22 (0.22, 0.22)		0.22 (0.22, 0.22)	
	4	0.22 (0.22, 0.23)		0.22 (0.22, 0.23)		0.22 (0.22, 0.23)		0.22 (0.22, 0.23)	
	5 Lowest BW	0.23 (0.22, 0.23)	<0.0001	0.23 (0.22, 0.23)	<0.0001	0.23 (0.22, 0.23)	<0.0001	0.23 (0.23, 0.23)	<0.0001
C-reactive protein (nmol/l)	1 Highest BW	4.95 (4.48, 5.46)		4.67 (4.23, 5.15)		4.80 (4.40, 5.24)		4.54 (4.16, 4.95)	
	2	4.95 (4.47, 5.48)		4.80 (4.34, 5.31)		4.71 (4.30, 5.15)		4.57 (4.18, 5.00)	
	3	5.08 (4.61, 5.59)		5.06 (4.60, 5.57)		5.10 (4.68, 5.56)		5.09 (4.67, 5.53)	
	4	4.59 (4.16, 5.07)		4.74 (4.30, 5.22)		4.61 (4.22, 5.03)		4.75 (4.36, 5.18)	
	5 Lowest BW	4.73 (4.28, 5.22)	0.28	5.01 (4.54, 5.53)	0.42	5.07 (4.64, 5.53)	0.53	5.36 (4.91, 5.85)	0.01
Triacylglycerol (mmol/l)	1 Highest BW	0.79 (0.76, 0.81)		0.78 (0.75, 0.80)		0.78 (0.76, 0.81)		0.77 (0.75, 0.79)	
	2	0.83 (0.80, 0.85)		0.82 (0.79, 0.84)		0.82 (0.80, 0.84)		0.81 (0.79, 0.84)	
	3	0.80 (0.78, 0.83)		0.80 (0.78, 0.82)		0.80 (0.78, 0.82)		0.80 (0.78, 0.82)	
	4	0.81 (0.79, 0.84)		0.82 (0.80, 0.85)		0.81 (0.79, 0.84)		0.82 (0.80, 0.84)	
	5 Lowest BW	0.81 (0.79, 0.84)	0.26	0.82 (0.80, 0.85)	0.01	0.82 (0.80, 0.84)	0.04	0.83 (0.81, 0.86)	<0.001
HDL-cholesterol (mmol/l)	1 Highest BW	1.47 (1.45, 1.50)		1.49 (1.46, 1.51)		1.48 (1.46, 1.50)		1.49 (1.47, 1.51)	
	2	1.48 (1.46, 1.51)		1.49 (1.47, 1.51)		1.49 (1.47, 1.51)		1.50 (1.47, 1.52)	
	3	1.48 (1.46, 1.51)		1.48 (1.46, 1.51)		1.48 (1.46, 1.51)		1.48 (1.46, 1.51)	
	4	1.49 (1.47, 1.51)		1.48 (1.46, 1.50)		1.49 (1.47, 1.51)		1.48 (1.46, 1.50)	
	5 Lowest BW	1.50 (1.47, 1.52)	0.17	1.48 (1.46, 1.50)	0.57	1.49 (1.47, 1.51)	0.56	1.47 (1.45, 1.50)	0.17
LDL-cholesterol (mmol/l)	1 Highest BW	2.62 (2.57, 2.67)		2.63 (2.58, 2.69)		2.62 (2.57, 2.67)		2.63 (2.58, 2.68)	
	2	2.65 (2.60, 2.71)		2.66 (2.61, 2.71)		2.65 (2.60, 2.70)		2.65 (2.60, 2.71)	
	3	2.58 (2.54, 2.63)		2.59 (2.54, 2.63)		2.59 (2.54, 2.63)		2.59 (2.54, 2.63)	
	4	2.60 (2.55, 2.65)		2.59 (2.54, 2.64)		2.60 (2.55, 2.65)		2.59 (2.54, 2.64)	
	5 Lowest BW	2.63 (2.58, 2.68)	0.53	2.61 (2.56, 2.67)	0.17	2.63 (2.58, 2.68)	0.76	2.62 (2.57, 2.67)	0.29
Systolic BP (mmHg)[a]	1 Highest BW	105.3 (104.5, 106.1)		104.4 (103.6, 105.2)		105.2 (104.4, 106.0)		104.4 (103.6, 105.2)	
	2	105.6 (104.7, 106.4)		105.1 (104.3, 106.0)		105.4 (104.6, 106.3)		105.0 (104.2, 105.8)	
	3	105.0 (104.2, 105.8)		104.9 (104.1, 105.7)		105.0 (104.2, 105.8)		104.9 (104.2, 105.7)	
	4	104.2 (103.3, 105.0)		104.6 (103.8, 105.4)		104.2 (103.4, 105.0)		104.6 (103.8, 105.4)	
	5 Lowest BW	104.2 (103.4, 105.1)	0.004	105.1 (104.3, 105.9)	0.52	104.4 (103.6, 105.3)	0.02	105.3 (104.5, 106.1)	0.21
Diastolic BP (mmHg)[a]	1 Highest BW	63.0 (62.2, 63.7)		62.6 (61.9, 63.4)		62.9 (62.1, 63.6)		62.6 (61.8, 63.3)	
	2	63.3 (62.6, 64.1)		63.2 (62.4, 63.9)		63.2 (62.4, 64.0)		63.0 (62.3, 63.8)	
	3	63.1 (62.4, 63.9)		63.1 (62.4, 63.8)		63.1 (62.4, 63.9)		63.1 (62.4, 63.8)	
	4	62.5 (61.8, 63.3)		62.7 (61.9, 63.5)		62.5 (61.8, 63.3)		62.7 (62.0, 63.4)	
	5 Lowest BW	62.8 (62.1, 63.6)	0.33	63.1 (62.4, 63.9)	0.62	63.0 (62.2, 63.7)	0.66	63.3 (62.6, 64.1)	0.31

[a] Absolute differences in blood pressure are presented.
Percentage differences in outcome are presented for log transformed variables (all except blood pressure).
Standard adjustment is for sex, age (in fourths), ethnic sub-group, NS-SEC group, height and a random effect for school.
Mean birth weight in each birth weight group: 1 = 3985g , 2 = 3540g, 3 = 3270g, 4 = 2990g, 5 = 2438g.
Abbreviations: BP, blood pressure; BW, birth weight.
