# Supplementary material for: Birthweight and risk markers for type 2 diabetes and cardiovascular disease in childhood: the Child Heart and Health Study in England (CHASE)
Source: Diabetologia. 2014 Dec 18;58(3):474–84. doi: 10.1007/s00125-014-3474-7 (PMC4320299; doi:10.1007/s00125-014-3474-7)
Supplement: Supplementary file 4 — (PDF 42 kb) [file 125_2014_3474_MOESM4_ESM.pdf]

ESM Table 4: Associations between birth weight and risk markers for type 2 diabetes and cardiovascular disease with additional adjustment for gestational age and parity

Blood analytes (N = 3114)	Adjustment for gestational age and parity?	% Difference/difference in outcome for a 100g increase in birth weight (95% CI), p-value		
Insulin (pmol/l)	No	-0.53	(-0.88, -0.16)	0.005
	GA (weeks)	-0.55	(-0.97, -0.11)	0.01
	GA + parity	-0.63	(-1.05, -0.19)	0.005
HOMA-IR	No	-0.56	(-0.91, -0.20)	0.002
	GA (weeks)	-0.58	(-0.99, -0.14)	0.009
	GA + parity	-0.66	(-1.08, -0.23)	0.003
HbA1c (%)	No	-0.04	(-0.08, 0.00)	0.03
	GA (weeks)	-0.07	(-0.12, -0.02)	0.003
	GA + parity	-0.07	(-0.12, -0.03)	0.002
HbA1c (mmol/l)	No	-0.07	(-0.14, 0.00)	0.04
	GA (weeks)	-0.12	(-0.20, -0.04)	0.004
	GA + parity	-0.12	(-0.21, -0.04)	0.003
Glucose (mmol/l)	No	-0.08	(-0.12, -0.03)	0.002
	GA (weeks)	-0.05	(-0.11, 0.01)	0.10
	GA + parity	-0.06	(-0.11, 0.00)	0.07
Urate (mmol/l)	No	-0.55	(-0.70, -0.40)	<0.0001
	GA (weeks)	-0.52	(-0.70, -0.34)	<0.0001
	GA + parity	-0.50	(-0.68, -0.32)	<0.0001
C-reactive protein (nmol/l)	No	-0.32	(-1.10, 0.52)	0.44
	GA (weeks)	-0.25	(-1.18, 0.78)	0.62
	GA + parity	-0.13	(-1.09, 0.92)	0.79
Triacylglycerol (mmol/l)	No	-0.35	(-0.58, -0.12)	0.004
	GA (weeks)	-0.24	(-0.52, 0.04)	0.09
	GA + parity	-0.21	(-0.50, 0.08)	0.15
HDL-cholesterol (mmol/l)	No	0.04	(-0.09, 0.17)	0.58
	GA (weeks)	0.00	(-0.15, 0.16)	0.98
	GA + parity	-0.01	(-0.17, 0.15)	0.89
LDL-cholesterol (mmol/l)	No	0.08	(-0.08, 0.24)	0.32
	GA (weeks)	0.12	(-0.07, 0.31)	0.23
	GA + parity	0.11	(-0.08, 0.31)	0.25
Systolic BP (mmHg)[a]	No	-0.05	(-0.12, 0.01)	0.13
	GA (weeks)	-0.02	(-0.10, 0.06)	0.65
	GA + parity	-0.03	(-0.11, 0.05)	0.46
Diastolic BP (mmHg)[a]	No	-0.02	(-0.08, 0.04)	0.56
	GA (weeks)	0.00	(-0.07, 0.07)	0.93
	GA + parity	-0.01	(-0.08, 0.07)	0.86

[a] Absolute differences in blood pressure are presented.
Percentage differences in outcome are presented for log transformed variables (all except blood pressure).
All associations are adjusted for sex, age (in fourths), ethnic sub-group, NS-SEC group, height and a random effect for school.
Abbreviations: BP, blood pressure; CI, confidence interval; GA, gestational age.
