# Supplementary material for: Birthweight and risk markers for type 2 diabetes and cardiovascular disease in childhood: the Child Heart and Health Study in England (CHASE)
Source: Diabetologia. 2014 Dec 18;58(3):474–84. doi: 10.1007/s00125-014-3474-7 (PMC4320299; doi:10.1007/s00125-014-3474-7)
Supplement: Supplementary file 5 — (PDF 42 kb) [file 125_2014_3474_MOESM5_ESM.pdf]

ESM Table 5: Associations between birth weight and risk markers for type 2 diabetes and cardiovascular disease before and after the removal of participants whose mother experienced gestational diabetes in pregnancy or participants who only had birth weight data available from parental recall

| Blood analytes (N = 3744) | Exclusions | % Difference/difference in outcome for a 100g increase in birth weight (95% CI), p-value | | |
|---|---|---|---|---|
| Insulin (pmol/l) | None | -0.41 | (-0.74, -0.08) | 0.02 |
| | Gestational diabetes (n=94) | -0.40 | (-0.72, -0.06) | 0.02 |
| | Parental recall of BW (n=512) | -0.44 | (-0.76, -0.11) | 0.01 |
| HOMA-IR | None | -0.42 | (-0.75, -0.09) | 0.01 |
| | Gestational diabetes (n=94) | -0.44 | (-0.79, -0.07) | 0.02 |
| | Parental recall of BW (n=512) | -0.04 | (-0.08, 0.00) | 0.03 |
| HbA1c (%) | None | -0.05 | (-0.08, -0.01) | 0.01 |
| | Gestational diabetes (n=94) | -0.04 | (-0.08, 0.00) | 0.04 |
| | Parental recall of BW (n=512) | -0.07 | (-0.13, 0.00) | 0.03 |
| HbA1c (%) | None | -0.08 | (-0.14, -0.01) | 0.02 |
| | Gestational diabetes (n=94) | -0.07 | (-0.14, 0.00) | 0.05 |
| | Parental recall of BW (n=512) | -0.06 | (-0.10, -0.02) | 0.01 |
| Glucose (mmol/l) | None | -0.06 | (-0.11, -0.02) | 0.01 |
| | Gestational diabetes (n=94) | -0.07 | (-0.12, -0.02) | 0.01 |
| | Parental recall of BW (n=512) | -0.52 | (-0.66, -0.38) | <0.0001 |
| Urate (mmol/l) | None | -0.52 | (-0.65, -0.38) | <0.0001 |
| | Gestational diabetes (n=94) | -0.54 | (-0.69, -0.40) | <0.0001 |
| | Parental recall of BW (n=512) | -0.23 | (-0.94, 0.55) | 0.56 |
| C-reactive protein (nmol/l) | None | -0.18 | (-0.92, 0.61) | 0.64 |
| | Gestational diabetes (n=94) | -0.30 | (-0.51, -0.09) | 0.01 |
| | Parental recall of BW (n=512) | -0.28 | (-0.49, -0.06) | 0.01 |
| Triacylglycerol (mmol/l) | None | -0.31 | (-0.54, -0.08) | 0.01 |
| | Gestational diabetes (n=94) | 0.04 | (-0.08, 0.16) | 0.53 |
| | Parental recall of BW (n=512) | 0.03 | (-0.09, 0.15) | 0.63 |
| HDL-cholesterol (mmol/l) | None | 0.03 | (-0.09, 0.16) | 0.60 |
| | Gestational diabetes (n=94) | 0.12 | (-0.02, 0.27) | 0.10 |
| | Parental recall of BW (n=512) | 0.15 | (0.00, 0.30) | 0.06 |
| LDL-cholesterol (mmol/l) | None | 0.10 | (-0.06, 0.26) | 0.22 |
| | Gestational diabetes (n=94) | -0.03 | (-0.09, 0.03) | 0.32 |
| | Parental recall of BW (n=512) | -0.03 | (-0.09, 0.03) | 0.31 |
| Systolic BP (mmHg)[a] | None | -0.04 | (-0.11, 0.02) | 0.22 |
| | Gestational diabetes (n=94) | -0.02 | (-0.07, 0.04) | 0.55 |
| | Parental recall of BW (n=512) | -0.01 | (-0.07, 0.04) | 0.60 |
| Diastolic BP (mmHg)[a] | None | -0.04 | (-0.10, 0.02) | 0.22 |
| | Gestational diabetes (n=94) | -0.41 | (-0.76, -0.04) | 0.03 |
| | Parental recall of BW (n=512) | -0.17 | (-0.95, 0.68) | 0.69 |

[a] Absolute differences in blood pressure are presented.
Percentage differences in outcome are presented for log transformed variables (all except blood pressure).
All models are adjusted for sex, age (in fourths), ethnic sub-group, NS-SEC group, height and a random effect for school. Exclusions: n=number of participants excluded.
Abbreviations: BP, blood pressure; CI, confidence interval.
