# Supplementary material for: Birthweight and risk markers for type 2 diabetes and cardiovascular disease in childhood: the Child Heart and Health Study in England (CHASE)
Source: Diabetologia. 2014 Dec 18;58(3):474–84. doi: 10.1007/s00125-014-3474-7 (PMC4320299; doi:10.1007/s00125-014-3474-7)
Supplement: Supplementary file 6 — (PDF 45 kb) [file 125_2014_3474_MOESM6_ESM.pdf]

ESM Table 6: Ethnic differences between South Asians and white Europeans in risk markers for type 2 diabetes and cardiovascular disease: effect of adjustment for birth weight

		% Difference/difference (95% Confidence Interval), p-value											
	Adjustment				South Asian sub-groups								
Outcome	for BW?	South Asian - white European			Indian - white European			Pakistani - white European			Bangladeshi - white European		
Insulin (pmol/l)	No	31.98	(24.71, 39.68)	<0.0001	32.26	(22.02, 43.36)	<0.0001	24.48	(15.15, 34.57)	<0.0001	48.05	(35.10, 62.24)	<0.0001
	Yes	32.71	(25.34, 40.52)	<0.0001	33.19	(22.81, 44.43)	<0.0001	25.18	(15.75, 35.36)	<0.0001	48.80	(35.76, 63.09)	<0.0001
HOMA-IR	No	31.55	(24.38, 39.14)	<0.0001	31.23	(21.17, 42.13)	<0.0001	24.65	(15.40, 34.65)	<0.0001	47.75	(34.96, 61.75)	<0.0001
	Yes	32.17	(24.90, 39.86)	<0.0001	32.01	(21.83, 43.04)	<0.0001	25.24	(15.91, 35.33)	<0.0001	48.38	(35.51, 62.48)	<0.0001
HbA1c (%)	No	2.06	(1.48, 2.65)	<0.0001	2.70	(1.87, 3.53)	<0.0001	2.64	(1.84, 3.45)	<0.0001	0.65	(-0.27, 1.58)	0.17
	Yes	2.00	(1.42, 2.59)	<0.0001	2.62	(1.79, 3.46)	<0.0001	2.59	(1.78, 3.39)	<0.0001	0.60	(-0.33, 1.53)	0.21
HbA1c (mmol/l)	No	3.46	(2.43, 4.49)	<0.0001	4.55	(3.08, 6.03)	<0.0001	4.50	(3.08, 5.93)	<0.0001	0.95	(-0.66, 2.58)	0.25
	Yes	3.35	(2.32, 4.40)	<0.0001	4.43	(2.95, 5.92)	<0.0001	4.40	(2.98, 5.84)	<0.0001	0.86	(-0.74, 2.50)	0.29
Glucose (mmol/l)	No	0.78	(0.07, 1.49)	0.03	1.08	(0.07, 2.11)	0.04	1.03	(0.05, 2.02)	0.04	1.12	(-0.03, 2.28)	0.06
	Yes	0.71	(-0.01, 1.43)	0.05	1.00	(-0.01, 2.03)	0.05	0.97	(-0.01, 1.96)	0.05	1.06	(-0.08, 2.23)	0.07
Urate (mmol/l)	No	0.84	(-1.38, 3.12)	0.46	-2.27	(-5.32, 0.88)	0.16	-1.37	(-4.34, 1.69)	0.38	7.92	(4.15, 11.82)	<0.0001
	Yes	0.01	(-2.21, 2.28)	0.99	-3.17	(-6.20, -0.04)	0.05	-2.12	(-5.07, 0.92)	0.17	7.11	(3.37, 10.99)	<0.001
C-reactive protein (nmol/l)	No	53.30	(36.23, 72.51)	<0.0001	43.01	(20.84, 69.25)	<0.0001	68.47	(43.20, 98.20)	<0.0001	50.49	(24.51, 81.89)	<0.0001
	Yes	54.77	(37.38, 74.36)	<0.0001	44.69	(22.13, 71.42)	<0.0001	70.09	(44.47, 100.25)	<0.0001	51.88	(25.59, 83.66)	<0.0001
Triacylglycerol (mmol/l)	No	13.21	(9.38, 17.18)	<0.0001	11.00	(5.69, 16.57)	<0.0001	13.68	(8.42, 19.20)	<0.0001	18.29	(11.91, 25.03)	<0.0001
	Yes	12.85	(8.99, 16.83)	<0.0001	10.62	(5.30, 16.22)	<0.0001	13.38	(8.11, 18.90)	<0.0001	17.99	(11.61, 24.73)	<0.0001
HDL-cholesterol (mmol/l)	No	-1.98	(-3.76, -0.16)	0.033	-0.06	(-2.64, 2.59)	0.97	-1.17	(-3.63, 1.36)	0.363	-5.68	(-8.42, -2.86)	<0.0001
	Yes	-2.13	(-3.93, -0.30)	0.02	-0.24	(-2.84, 2.42)	0.86	-1.31	(-3.79, 1.22)	0.307	-5.82	(-8.56, -2.99)	<0.0001
LDL-cholesterol (mmol/l)	No	3.92	(1.60, 6.29)	<0.001	3.93	(0.65, 7.32)	0.02	4.66	(1.47, 7.96)	0.004	1.96	(-1.67, 5.72)	0.29
	Yes	4.06	(1.71, 6.46)	<0.001	4.11	(0.80, 7.53)	0.01	4.81	(1.60, 8.12)	0.003	2.09	(-1.55, 5.87)	0.26
Systolic BP (mmHg)[a]	No	-0.84	(-1.79, 0.12)	0.09	-0.56	(-1.92, 0.80)	0.42	-0.95	(-2.26, 0.37)	0.16	-0.92	(-2.46, 0.61)	0.24
	Yes	-0.69	(-1.65, 0.27)	0.16	-0.38	(-1.75, 0.98)	0.58	-0.80	(-2.12, 0.52)	0.23	-0.79	(-2.33, 0.75)	0.32
Diastolic BP (mmHg)[a]	No	1.13	(0.27, 1.98)	0.01	1.72	(0.50, 2.94)	0.01	0.48	(-0.70, 1.66)	0.43	1.27	(-0.11, 2.65)	0.07
	Yes	1.17	(0.31, 2.03)	0.01	1.77	(0.55, 3.00)	0.00	0.52	(-0.66, 1.70)	0.39	1.31	(-0.08, 2.70)	0.06

[a] Absolute differences in blood pressure are presented. Percentage differences in outcome are presented for log transformed variables (all except blood pressure).
Standard adjustment is for sex, age (in fourths), NS-SEC group and a random effect for school.
Abbreviations: BP, blood pressure; BW, birth weight; CI, confidence interval.
