# Supplementary material for: Birthweight and risk markers for type 2 diabetes and cardiovascular disease in childhood: the Child Heart and Health Study in England (CHASE)
Source: Diabetologia. 2014 Dec 18;58(3):474–84. doi: 10.1007/s00125-014-3474-7 (PMC4320299; doi:10.1007/s00125-014-3474-7)
Supplement: Supplementary file 7 — (PDF 44 kb) [file 125_2014_3474_MOESM7_ESM.pdf]

ESM Table 7: Ethnic differences between black African-Caribbeans and white Europeans in risk markers for type 2 diabetes and cardiovascular disease: effect of adjustment for birth weight

		% Difference/difference (95% Confidence Interval), p-value								
	Adjustment	Black African-Caribbean - white European			Black African-Caribbean sub-groups					
Outcome	for BW?				Black Caribbean - white European			Black African - white European		
Insulin (pmol/l)	No	24.66	(17.68, 32.05)	<0.0001	30.99	(21.49, 41.23)	<0.0001	21.69	(13.41, 30.58)	<0.0001
	Yes	24.86	(17.87, 32.27)	<0.0001	31.43	(21.88, 41.73)	<0.0001	21.75	(13.46, 30.64)	<0.0001
HOMA-IR	No	24.13	(17.25, 31.41)	<0.0001	30.36	(21.00, 40.45)	<0.0001	21.45	(13.26, 30.22)	<0.0001
	Yes	24.30	(17.41, 31.59)	<0.0001	30.74	(21.33, 40.87)	<0.0001	21.49	(13.30, 30.27)	<0.0001
HbA1c (%)	No	1.74	(1.15, 2.33)	<0.0001	1.59	(0.83, 2.37)	<0.0001	1.96	(1.24, 2.69)	<0.0001
	Yes	1.72	(1.14, 2.31)	<0.0001	1.56	(0.79, 2.33)	<0.0001	1.96	(1.24, 2.68)	<0.0001
HbA1c (mmol/l)	No	2.82	(1.79, 3.86)	<0.0001	2.55	(1.20, 3.92)	<0.001	3.20	(1.94, 4.49)	<0.0001
	Yes	2.79	(1.76, 3.83)	<0.0001	2.50	(1.15, 3.86)	<0.001	3.20	(1.93, 4.48)	<0.0001
Glucose (mmol/l)	No	-0.71	(-1.42, 0.01)	0.05	-0.50	(-1.44, 0.44)	0.29	-0.41	(-1.28, 0.47)	0.36
	Yes	-0.73	(-1.44, -0.01)	0.05	-0.54	(-1.47, 0.40)	0.26	-0.42	(-1.29, 0.46)	0.35
Urate (mmol/l)	No	-6.57	(-8.70, -4.39)	<0.0001	-3.91	(-6.77, -0.96)	0.01	-8.29	(-10.84, -5.67)	<0.0001
	Yes	-6.81	(-8.93, -4.64)	<0.0001	-4.36	(-7.20, -1.42)	0.004	-8.37	(-10.91, -5.76)	<0.0001
C-reactive protein (nmol/l)	No	24.46	(10.21, 40.56)	<0.001	36.37	(16.22, 60.02)	<0.001	23.29	(6.21, 43.11)	0.01
	Yes	24.83	(10.52, 40.98)	<0.001	37.16	(16.86, 60.98)	<0.001	23.41	(6.32, 43.25)	0.01
Triacylglycerol (mmol/l)	No	-10.55	(-13.64, -7.35)	<0.0001	-7.32	(-11.50, -2.95)	0.00	-12.71	(-16.39, -8.87)	<0.0001
	Yes	-10.63	(-13.72, -7.43)	<0.0001	-7.47	(-11.65, -3.10)	<0.001	-12.73	(-16.41, -8.89)	<0.0001
HDL-cholesterol (mmol/l)	No	2.39	(0.48, 4.35)	0.01	1.39	(-1.10, 3.94)	0.28	2.59	(0.24, 4.99)	0.03
	Yes	2.35	(0.43, 4.30)	0.02	1.30	(-1.19, 3.85)	0.31	2.57	(0.22, 4.98)	0.03
LDL-cholesterol (mmol/l)	No	-1.23	(-3.49, 1.08)	0.29	2.72	(-0.34, 5.87)	0.08	-4.61	(-7.27, -1.88)	0.001
	Yes	-1.19	(-3.45, 1.12)	0.31	2.81	(-0.26, 5.97)	0.07	-4.60	(-7.26, -1.87)	0.001
Systolic BP (mmHg)[a]	No	-0.28	(-1.26, 0.69)	0.57	0.36	(-0.92, 1.64)	0.58	-0.87	(-2.07, 0.32)	0.15
	Yes	-0.24	(-1.21, 0.73)	0.63	0.45	(-0.83, 1.73)	0.49	-0.86	(-2.05, 0.34)	0.16
Diastolic BP (mmHg)[a]	No	0.73	(-0.14, 1.60)	0.10	0.59	(-0.56, 1.73)	0.31	0.81	(-0.26, 1.88)	0.14
	Yes	0.74	(-0.13, 1.61)	0.10	0.61	(-0.53, 1.76)	0.29	0.81	(-0.26, 1.88)	0.14

[a] Absolute differences in blood pressure are presented. Percentage differences in outcome are presented for log transformed variables (all except blood pressure).
Standard adjustment is for sex, age (in fourths), NS-SEC group and a random effect for school.
Abbreviations: BP, blood pressure; BW, birth weight; CI, confidence interval.
