# Supplementary material for: Birthweight and risk markers for type 2 diabetes and cardiovascular disease in childhood: the Child Heart and Health Study in England (CHASE)
Source: Diabetologia. 2014 Dec 18;58(3):474–84. doi: 10.1007/s00125-014-3474-7 (PMC4320299; doi:10.1007/s00125-014-3474-7)
Supplement: Supplementary file 8 — (PDF 47 kb) [file 125_2014_3474_MOESM8_ESM.pdf]

ESM Figure 1: Associations between birth weight (in fifths) and risk markers for type 2 diabetes and cardiovascular disease (means and 95% confidence intervals)

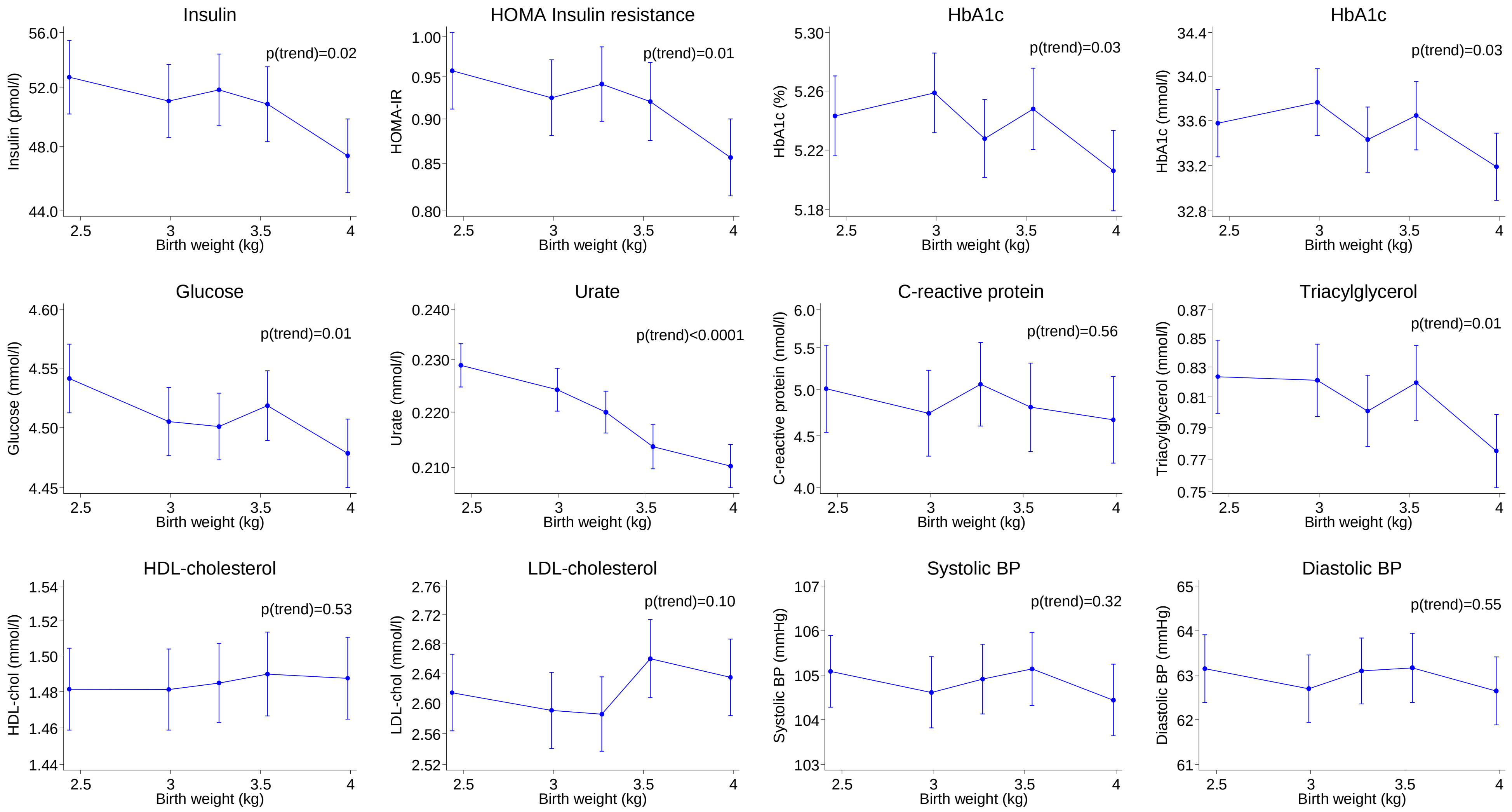

All models are adjusted for sex, age (in fourths), ethnic group, NS-SEC group, height and a random effect for school. All outcome variables are presented on the log scale (except for blood pressure and estimates are presented at the mean level of each birth weight quintile. P-values are presented for linear trend with birth weight as a continuous variable.
