# Supplementary material for: Birthweight and risk markers for type 2 diabetes and cardiovascular disease in childhood: the Child Heart and Health Study in England (CHASE)
Source: Diabetologia. 2014 Dec 18;58(3):474–84. doi: 10.1007/s00125-014-3474-7 (PMC4320299; doi:10.1007/s00125-014-3474-7)
Supplement: Supplementary file 9 — (PDF 63 kb) [file 125_2014_3474_MOESM9_ESM.pdf]

ESM Acknowledgments

We are grateful to staff at the following NHS Hospital Trusts which provided access to maternal health data in support of this research:-

Barking, Havering & Redbridge University Hospitals NHS Trust (King George's Hospital)

Barts Health NHS Trust (Whipps Cross, Newham & Royal London Hospitals)

Birmingham Women's NHS Foundation Trust (Birmingham Women & QE Hospitals)

Croydon University Hospital (Mayday Hospital)

Ealing Hospital NHS Trust (Ealing Hospital)

Epsom & St Helier University Hospital NHS Trust (St Helier Hospital)

Heart of England NHS Foundation Trust (Solihull, Good Hope & Heartlands Hospitals)

Homerton University Hospital NHS Foundation Trust (Homerton Hospital)

Imperial College Health Care NHS Trust (St Mary's, Hammersmith & Queen Charlotte Hospitals)

Kings College Hospital Foundation Trust (Kings College Hospital)

Leicester Hospital NHS Trust (Leicester Royal Infirmary & Leicester General Hospitals)

Lewisham & Greenwich NHS Trust (Lewisham Hospital)

North Middlesex Hospital NHS Trust (North Middlesex Hospital)

Royal Free London NHS Foundation Trust (Royal Free Hospital)

Sandwell & West Birmingham Hospitals NHS Trust (Birmingham City Hospital)

St George's Healthcare NHS Trust (St George's Hospital)

University College London Hospital Foundation NHS Trust (University College Hospital)

Whittington Health NHS (Whittington Hospital)
